# Expression of SMURF family in pancreatic cancer and its effect on cell proliferation and migration

**DOI:** 10.3389/fonc.2025.1538335

**Published:** 2025-04-24

**Authors:** Chen Zhang, Caoyi Li, Long Wu, Yuan Wang, Ke Huang, Xu Yang, Sai Zhao, Haoran Zhu, Lin Shi, Jian Wang

**Affiliations:** ^1^ Department of General Surgery, The Second Affiliated Hospital of Xuzhou Medical University, Xuzhou, Jiangsu, China; ^2^ Internal Medicine-Cardiovascular Department, Jiangsu University Affiliated Hospital, Zhenjiang, Jiangsu, China; ^3^ Cancer Institute, Xuzhou Medical University, Xuzhou, Jiangsu, China; ^4^ Jiangsu Provincial People’s Hospital, Nanjing, Jiangsu, China

**Keywords:** pancreatic cancer, SMURF, prognosis, proliferation, migration

## Abstract

**Objective:**

This study aims to evaluate the expression of Smad ubiquitination regulatory factors (SMURFs) in pancreatic cancer and analyze their relationship with cancer staging and prognosis, and to investigate the potential of SMURF as a therapeutic target for pancreatic cancer.

**Methods:**

A total of 179 patients with pancreatic cancer were identified in The Cancer Genome Atlas (TCGA) database. This dataset was utilized in the study to analyze the expression of SMURF1 and SMURF2 and their correlation with pancreatic cancer staging and patient survival. *In vitro* assays including CCK-8, EdU, colony formation, and wound-healing were employed to elucidate the function of SMURF1 in the proliferation and migration of pancreatic cancer cells.

**Results:**

High expression of SMURF1 showed a significant positive correlation with T staging, histological and pathological grades, as well as clinical treatment outcomes of pancreatic cancer (*P*<0.050). Meanwhile, high expression of SMURF2 indicated a positive correlation with the histological grade of pancreatic cancer (*P*<0.050). However, high expression of SMURF1 was negatively correlated with overall survival (OS) and progression-free interval (PFI) (*P*<0.050). High expression of SMURF2 was negatively correlated with PFI (*P*<0.050). Inhibition of SMURF1 expression suppressed the proliferation and migration of pancreatic cancer cells.

**Conclusion:**

High expression of SMURF1 could potentially be a therapeutic target and a poor prognostic indicator in pancreatic cancer.

## 1 Background

Pancreatic adenocarcinoma (PAC) is the 12th most common malignant tumor worldwide and one of the most invasive malignant tumors (1). The incidence and mortality of PAC are increasing, and it is expected to become the second leading cause of cancer-related deaths by 2030 (2). It is reported that the incidence rate of pancreatic cancer continues to rise, and it is currently the third leading cause of cancer death for men and women. The prognosis of PAC patients is extremely poor, with a 5-year OS rate is only 13% according to standard treatment (3). Because of its highly aggressive characteristics and low survival rate, it continues to impose a significant global disease burden. The challenges in treating this condition effectively can be attributed to the absence of adequate screening and diagnostic tools, the anatomical depth of the pancreas, the complexity of obtaining tissue biopsies, and the limited efficacy of radiotherapy or chemotherapy (4, 5). The etiology of PAC is still unclear (6). Currently, there are no clinical features other than the clinical stage of the disease that can guide treatment decisions for pancreatic cancer (7). Exploring additional molecular markers related to PAC prognosis is of great clinical significance.

Ubiquitination is a post-translational modification that plays a role in many biological processes (8). The classical ubiquitin-proteasome system (UPS) comprises three major factors: ubiquitin-activating enzyme (E1), conjugating enzyme (E2), and protein ligase (E3) (9, 10). Micel LN et al. reported that UPS dysfunction contributes to the development of cancer, making it a feasible and rational target for new therapies (11). Numerous E3 ubiquitin ligases in the ubiquitin-proteasome pathway are linked to cell cycle regulation and uncontrolled cell proliferation. SMURF1 and SMURF2 are homologous to E6-associated protein C-terminus (HECT) E3 ubiquitin ligases that regulate TGF-β/BMP signaling through ubiquitination, which leads to protein degradation and thus prevents overactivation of TGF-β/BMP signaling (12). SMURF1 and SMURF2 exhibit high sequence homology and similar structural features (13). SMURF is a negative regulator in the transforming growth factor (TGF) signaling cascade. Studies have shown that SMURF has anti-tumor activity in prostate cancer, but it is important to note that this effect may be context-dependent (14, 15). Fan et al. reported that SMURF1 is involved in cell migration and invasion in various cancers, including breast cancer (16, 17), colon cancer (18), gastric cancer (19), lung cancer (20), adenoid cystic carcinoma, and salivary gland carcinoma (21). Longtao Yang et al. reported that SMURF1/2, as a tumor promoter, is overexpressed in tumor cells, leading to poor prognosis. SMURF1 promotes the migration of breast cancer cells through ubiquitination and RhoA degradation (22). Lin Fu et al. demonstrated through genomic hybridization experiments that SMURF1 is a potential carcinogen for a variety of cancers. The expression level of SMURF1 is negatively correlated with the survival rate in gastric cancer and renal clear cell carcinoma. Knocking out SMURF1 can reduce the occurrence of tumors in gastric cancer, prostate cancer, and ovarian cancer (23). Furthermore, high expression levels of SMURF2 are associated with a poor prognosis in esophageal cancer.

However, the prognostic significance of SMURF in pancreatic cancer is currently unclear. In this study, the association between SMURF presence, clinical grade of pancreatic cancer, and patient survival was investigated, with a focus on clarifying the impact of SMURF on the prognosis of pancreatic cancer patients and assessing its potential as a therapeutic target.

## 2 Patients and methods

### 2.1 Patients

In this study, information on 179 cancer patients was extracted from TCGA database on January 18, 2024 (https://portal.gdc.cancer.gov/). The expression data of SMURF family (SMURF1, SMURF2) were collected from these patients. All clinical information and survival data were acquired from the selected patients at the time of diagnosis. The clinical information at diagnosis was outlined, including age, gender, tumor TNM stage, pathological stage, histological grade, history of radiotherapy, primary treatment outcomes, race, smoking history, alcohol consumption history, history of diabetes, history of chronic pancreatitis, family history of tumors, OS, PFI, and disease-specific survival (DSS), excluding specific treatment methods for patients. OS, PFI, and DSS were used as endpoints. All patient-related information, including clinical data, molecular data, and microarray datasets, was available from TCGA. The pancreatic cancer tissues and adjacent tissues of five cases were obtained from the pancreatic biobank, the First Affiliated Hospital with Nanjing Medical University {Sample collection time: 2020-2022. Patient age: 54-73 years old. Median age: 64 years old. Inclusion criteria of patients: patients with pancreatic cancer who were newly treated; Age ≥ 18 years old; Surgical treatment is feasible for primary and metastatic pancreatic tumors, and surgical treatment is planned upon admission; Exclusion criteria for patients: neoadjuvant therapy (radiotherapy, chemotherapy, molecular targeted therapy, immunotherapy, etc.) has been performed before surgery; Patients with low intelligence or dementia (unable to cooperate with this study) or unwilling to sign the informed consent form for this clinical study.} This study obtained the patients’ written informed consent and was approved by The Second Affiliated Hospital of Xuzhou Medical University (also known as Xuzhou Mining Group General Hospital). All samples were frozen in liquid nitrogen.

### 2.2 Methods

#### 2.2.1 Cell lines, antibodies and inhibitors

The PANC-1 and CFPAC-1 cell lines in this study were purchased from Hunan FengHui Biotechnology Co., Ltd. and cultured at 37°C in DMEM medium containing 5% CO2, 10% FBS, 1μg/mL penicillin and streptomycin. CFPAC-1 cells are a pancreatic ductal adenocarcinoma cell line that expresses characteristic cytokeratin and carcinoembryonic antigen of pancreatic ductal cells. PANC-1 cells were derived from pancreatic duct tissue of a patient with pancreatic cancer and has epithelial cell morphology. SMURF1 (ab57573, 1:2000) primary antibodies were purchased from ABCAM, Cyclin B1 (GB11255, 1:1000), Cyclin D1 (GB111372, 1:1000), and β-ACTIN (GB15003, 1:1000) were purchased from Servicebio. Smurf1-IN-A01 (A01) (T16904) (24) is an inhibitor of the ubiquitin ligase Smad ubiquitination regulatory factor-1 (SMURF1), with a kd of 3.664 nM. It can inhibit Smad1/5 degradation mediated by SMURF1 and enhance BMP-2 reactivity. A01 purchased from TargetMol, and diluted to different concentrations in DMEM medium before use.

#### 2.2.2 Cell viability assay

For the determination of cell survival rate, PANC-1 and CFPAC-1 cell lines were first seeded on a 96 well plate at a density of 3000 cells per well and grown adherent for 24 hours. Cells were treated with 0.1% dimethyl sulfoxide (DMSO) or different doses (0-100 μM) of Smurf1-IN-A01 for 72 hours. Then 10 μL of CCK8 reagent was added to each well and incubated for 2 hours. Absorbance was measured at 490 nm on a microplate reader. The experiment was repeated three times.

#### 2.2.3 EdU incorporation assays

Cell-Light™ EdU Cell Proliferation Detection Kit was used to analyze cell proliferation. Cells were cultured overnight in a 96-well plate containing 6*10^3^ cells per well and then treated with different doses of Smurf1-IN-A01 (0, 20, and 40 μmol/L) for 24 hours, followed by incubation with 50 μmol/L EdU for 2 hours. After the incubation period, the cells were fixed with 4% paraformaldehyde for 30 minutes, and then treated with 0.5% Triton X-100 for 20 minutes. Subsequently, the samples were incubated in 1× Apollo^®^ Reaction cocktail for 30 minutes. The cellular DNA was stained with DAPI for 15 minutes and washed three times with PBS. Finally, the cells were analyzed using a fluorescence microscope (Olympus, Japan), and images were captured.

#### 2.2.4 Colony formation experiments

Cells were exposed to 0.1% DMSO (vector) or Smurf1-IN-A01 (0, 20, and 40 μmol/L) in 6-well plates containing 100 cells per well for 24 hours. The culture medium was renewed every 6 days during the colony formation period of 15-17 days. Next, the cells were fixed with 4% formaldehyde and then stained with 0.1% crystal violet solution. Positive colonies were then manually quantified.

#### 2.2.5 Wound-healing assay

Pancreatic cancer cells were spread in 6-well plates and cultured overnight. Once the cells reached 90% fusion, a plastic pipette tip was used to scratch the wound on a single layer of cells. After washing in PBS, the culture medium was replaced with serum-free medium containing 0.1% DMSO or different concentrations of Smurf1-IN-A01. The cells were then cultured for 24 or 48 hours, and five fields of view at the edge of the ruptured area were randomly selected for imaging under a microscope. This experiment was conducted three times. The number of cells migrating from the scratch in each treatment group was counted for statistical analysis.

#### 2.2.6 Western blot analysis

PANC-1 and CFPAC-1 cells were treated with Smurf1-IN-A01 (0, 20, and 40 μmol/L) in 6-well plates containing 5*10^6^ cells per well for 24 hours, and then total proteins were collected. For each sample, 50 µg of protein was separated by electrophoresis (10% SDS page gel) and then transferred to polyvinylidene fluoride (PVDF) membrane for further analysis. The membrane was blocked with 5% skim milk and incubated overnight with specific primary antibody at 4°C before being incubated with secondary antibody at room temperature for 2 hours. Expression levels of Smurf1, Cyclin D1, and Cyclin B1 were detected using specific antibodies with β-actin as a control.

### 2.3 Statistical analysis

OS, PFI, and DSS were used as endpoints. OS denotes the duration from diagnosis to death from any cause or the last follow-up. DSS refers to death brought on by a specific ailment. PFI indicates the duration of survival without further progression of the disease after treatment. Clinical and molecular characteristics of patients diagnosed with pancreatic cancer were summarized using descriptive statistics, and medians and/or ranges were reported. Categorical variables were tested by chi-square test. For numerical variables, T test was used for comparison between two groups, and One-way ANOVA was used for comparison between multiple groups. Dunnett t test was used to further compare each group. Spearman correlation analysis was used to analyze the correlation between SMURF1 and SMURF2. Survival analysis was performed by Kaplan-Meier method and log-rank test to illustrate the effect of SMURF1/2 expression on OS, PFI, and DSS. Multivariate Cox proportional hazards model analyses were conducted for OS, PFI, and DSS. The results were reported with a 95% confidence interval. A two-tailed P value of less than 0.05 was considered statistically significant for all analyses.

## 3 Results

### 3.1 Clinical and molecular characteristics

The clinical and molecular characteristics are outlined in **Table 1**. Based on data regarding colorectal cancer patients in the TCGA database, the samples were categorized according to the median expression level of SMURF gene. Samples with expression levels above the median were assigned to the SMURF high-expression group, while those below the median were assigned to the low-expression group (refer to **Table 1**). Due to some patients having missing clinical information, we excluded some of their clinical information from the statistics. The analysis revealed a higher proportion of the SMURF1 low-expression group in the T1 and T2 stages (high vs low: 4.5% vs 13%), while the proportion of the SMURF1 high-expression group was higher in T3 and T4 stages (high vs low: 46.3% vs 36.2%), and the difference was statistically significant (*P*=0.002). However, there was no statistically significant difference in SMURF2 expression in the T stage. In contrast, there was a statistically significant difference in SMURF1 expression in the pathological stage (*P*=0.034). For histological grade, the proportion of the SMURF1 low-expression group was higher in G1 and G2 stages (high vs low: 32.2% vs 39.5%), while the proportion of the SMURF1 high-expression group was higher in G3 and G4 stages (high vs low: 18.6% vs 9.6%), and there was a statistically significant difference (*P*=0.011). Similarly, SMURF2 expression showed statistically significant difference in histological grade (*P*=0.022), with a higher proportion of SMURF2 low-expression group in G1 and G2 stages (high vs low: 32.2% vs 39.5%), whereas the proportion of SMURF2 high-expression group was higher in G3 and G4 stages (high vs low: 18.1% vs 10.2%). In terms of primary treatment outcomes, it was found that in patients with progression of disease (PD), the proportion of the SMURF1 high-expression group was higher than that of the SMURF1 low-expression group (high vs low: 25.7% vs 10.0%). However, in patients with stable disease (SD), partial response (PR) and complete response (CR), the proportion of the SMURF1 low-expression group was higher (high vs low: 1.4% vs 5%, 2.9% vs 4.3% and 21.4% vs 29.3%, respectively), and the difference in SMURF1 expression was statistically significant (*P*=0.002).

**Table 1:** Characteristics of 179 patients with pancreatic cancer in the TCGA database.

Variable	SMURF1 (Low)	SMURF1 (High)	*P* Value	SMURF2 (Low)	SMURF2 (High)	*P* Value
n	89	90		89	90	
Pathologic T stage, n (%)	87	90	0.002	87	90	0.137
T1&T2	23 (13%)	8 (4.5%)		19 (10.7%)	12 (6.8%)	
T3&T4	64 (36.2%)	82 (46.3%)		68 (38.4%)	78 (44.1%)	
Pathologic N stage, n (%)	85	89	0.598	85	89	0.598
N0	26 (14.9%)	24 (13.8%)		26 (14.9%)	24 (13.8%)	
N1	59 (33.9%)	65 (37.4%)		59 (33.9%)	65 (37.4%)	
Pathologic M stage, n (%)	42	43	0.371	29	56	0.841
M0	41 (48.2%)	39 (45.9%)		28 (32.9%)	52 (61.2%)	
M1	1 (1.2%)	4 (4.7%)		1 (1.2%)	4 (4.7%)	
Pathologic stage, n (%)	87	89	0.034	86	90	0.140
Stage I	16 (9.1%)	5 (2.8%)		12 (6.8%)	9 (5.1%)	
Stage II	68 (38.6%)	79 (44.9%)		70 (39.8%)	77 (43.8%)	
Stage III	2 (1.1%)	1 (0.6%)		3 (1.7%)	0 (0%)	
Stage IV	1 (0.6%)	4 (2.3%)		1 (0.6%)	4 (2.3%)	
Histologic grade, n (%)	87	90	0.011	88	89	0.022
G1&G2	70 (39.5%)	57 (32.2%)		70 (39.5%)	57 (32.2%)	
G3&G4	17 (9.6%)	33 (18.6%)		18 (10.2%)	32 (18.1%)	
Primary therapy outcome, n (%)	68	72	0.002	68	72	0.197
PD	14 (10%)	36 (25.7%)		20 (14.3%)	30 (21.4%)	
SD	7 (5%)	2 (1.4%)		7 (5%)	2 (1.4%)	
PR	6 (4.3%)	4 (2.9%)		5 (3.6%)	5 (3.6%)	
CR	41 (29.3%)	30 (21.4%)		36 (25.7%)	35 (25%)	
Gender, n (%)	89	90	0.116	89	90	0.504
Female	45 (25.1%)	35 (19.6%)		42 (23.5%)	38 (21.2%)	
Male	44 (24.6%)	55 (30.7%)		47 (26.3%)	52 (29.1%)	
Age, n (%)	89	90	0.413	89	90	0.156
<= 65	44 (24.6%)	50 (27.9%)		42 (23.5%)	52 (29.1%)	
> 65	45 (25.1%)	40 (22.3%)		47 (26.3%)	38 (21.2%)	
History of diabetes, n (%)	73	74	0.670	79	68	0.330
No	53 (36.1%)	56 (38.1%)		56 (38.1%)	53 (36.1%)	
Yes	20 (13.6%)	18 (12.2%)		23 (15.6%)	15 (10.2%)	
History of chronic pancreatitis, n (%)	70	72	0.412	79	63	0.653
No	65 (45.8%)	64 (45.1%)		71 (50%)	58 (40.8%)	
Yes	5 (3.5%)	8 (5.6%)		8 (5.6%)	5 (3.5%)	
Alcohol history, n (%)	85	82	0.731	86	81	0.270
No	32 (19.2%)	33 (19.8%)		30 (18%)	35 (21%)	
Yes	53 (31.7%)	49 (29.3%)		56 (33.5%)	46 (27.5%)	

### 3.2 Relationship between survival time and expression of SMURF1 and SMURF2 in pancreatic cancer patients

**Figure 1:**
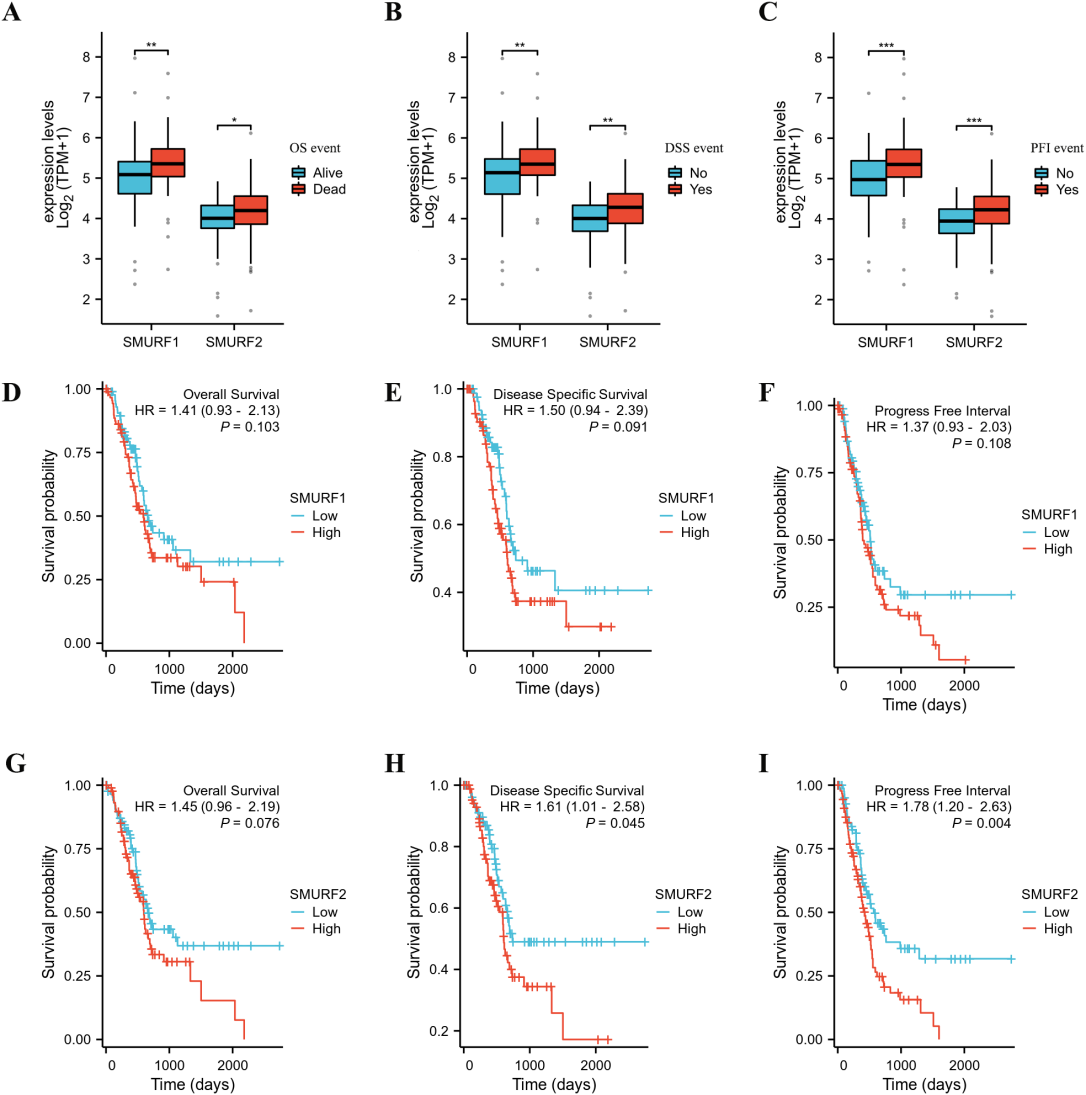
Kaplan-Meier curves for overall survival (OS), disease-specific survival (DSS), and progression-free interval (PFI) in the TCGA database. **(A–C)** The expression levels of SMURF1/2 are higher in deceased pancreatic cancer patients than in living ones. **(D–I)** Patients with low expression of SMURF2 have longer OS, DSS, and PFI than those with high expression. OS, overall survival; DSS, disease-specific survival; PFI, progression-free interval.

Patients with pancreatic cancer were divided into high and low expression groups based on the median expression levels of SMURF1 and SMURF2. Subsequently, OS, DSS, and PFI were compared (**Table 2**, **Figure 1**). Some patients have missing survival information, so we excluded some of their survival information from the statistics. The analysis demonstrated that the distribution of survival time between the groups was significantly different, as indicated by the results of the Cox regression analysis presented in **Figure 1**. All P-values were less than 0.05. Analysis and comparison of OS, DSS and PFI revealed that SMURF1 and SMURF2 were expressed at higher levels in deceased pancreatic cancer patients compared to living patients (**Figures 1A–C**). **Table 2** illustrates that high expression of SMURF1 may have a detrimental effect on OS and PFI, while high expression of SMURF2 only exhibits a negative impact on PFI. In terms of OS, the proportion of patients with low SMURF1 expression was greater than that of patients with high SMURF1 expression in the living cohort (high versus low: 20.1% versus 27.9%). Conversely, there were more patients with high than low SMURF1 expression in the deceased cohort (high vs. low: 30.2% vs. 21.8%). These differences were statistically significant (*P*=0.030). For PFI, the proportion of patients with low SMURF1 expression was higher than that of patients with high SMURF1 expression in the living cohort (high vs. low: 16.2% vs. 25.1%). In contrast, the proportion of patients with high SMURF1 expression exceeded that of patients with low SMURF1 expression in the living cohort (high versus low: 34.1% versus 24.6%), with a statistically significant difference (*P*=0.013). On the contrary, in terms of PFI, solely the SMURF2 expression exhibited statistically significant difference (*P*=0.005), accompanied by the proportion.

**Table 2:** Survival characteristics of 179 pancreatic cancer patients in the TCGA database.

Feature	SMURF1 (Low)	SMURF1 (High)	*P* Value	SMURF2 (Low)	SMURF2 (High)	*P* Value
n	89	90		89	90	
OS event, n (%)	89	90	0.030	89	90	0.117
Alive	50 (27.9%)	36 (20.1%)		48 (26.8%)	38 (21.2%)	
Dead	39 (21.8%)	54 (30.2%)		41 (22.9%)	52 (29.1%)	
DSS event, n (%)	86	87	0.053	85	88	0.071
No	56 (32.4%)	44 (25.4%)		55 (31.8%)	45 (26%)	
Yes	30 (17.3%)	43 (24.9%)		30 (17.3%)	43 (24.9%)	
PFI event, n (%)	89	90	0.013	89	90	0.005
No	45 (25.1%)	29 (16.2%)		46 (25.7%)	28 (15.6%)	
Yes	44 (24.6%)	61 (34.1%)		43 (24%)	62 (34.6%)	

OS, overall survival; DSS, disease-specific survival; PFI, progression-free interval.

### 3.3 Predictive role of SMURF in pancreatic cancer

**Figure 2:**
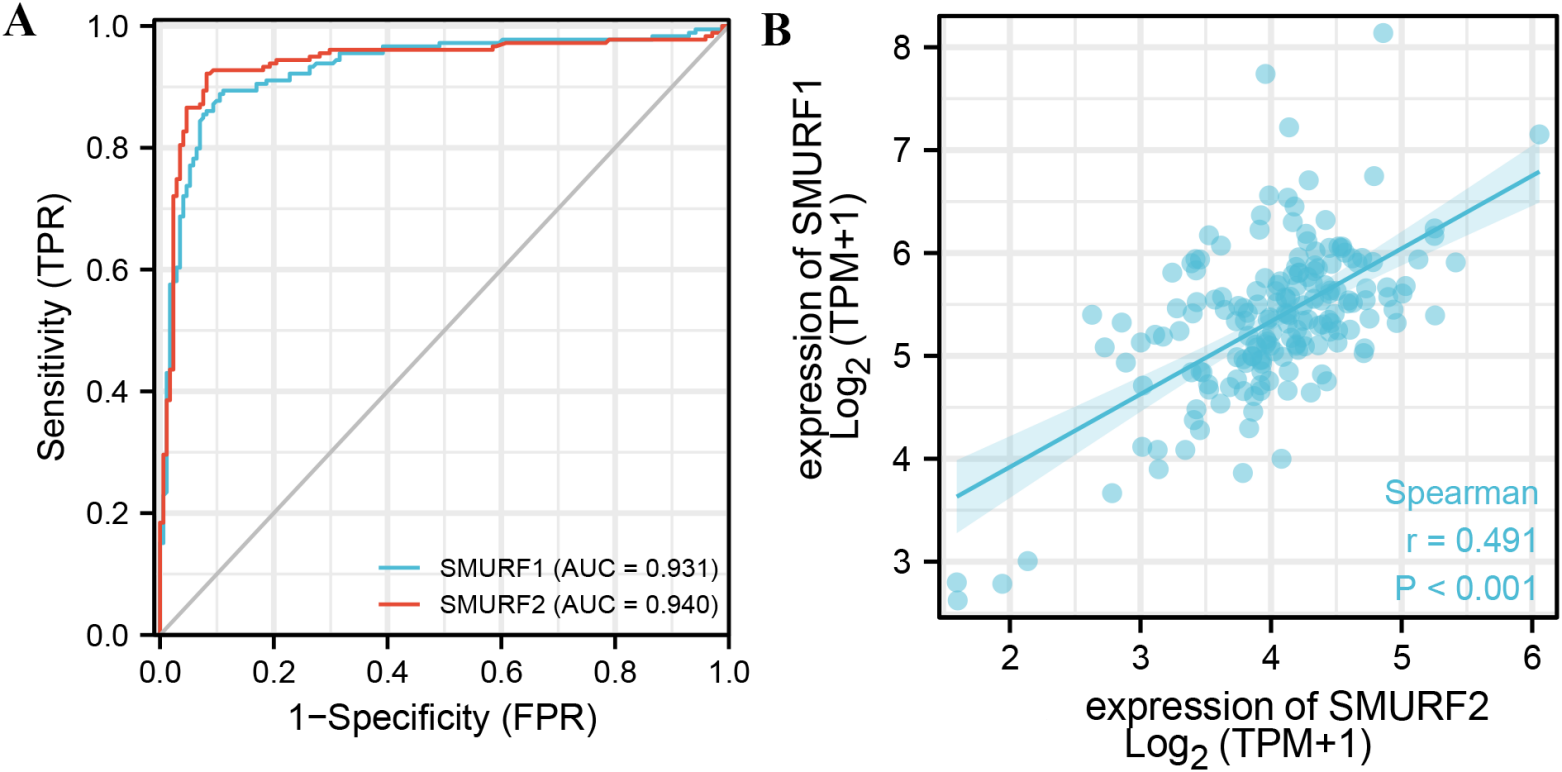
ROC curve of SMURF in pancreatic cancer and the correlation between the expression levels of SMURF members. **(A)** Indicates that the predictive ability of variables SMURF1 and SMURF2 has high accuracy in predicting normal and tumor prognosis. **(B)** shows the co-expression relationship between SMURF1 and SMURF2 genes in the TCGA database.

From **Table 3** and **Figure 2A**, it can be seen that variable SMURF1 had high accuracy in predicting outcomes between normal and tumor tissues (AUC = 0.931, CI = 0.902-0.960), and variable SMURF2 also had high accuracy in predicting outcomes (AUC = 0.940, CI = 0.911-0.969).

**Table 3:** ROC curve of SMURF in pancreatic cancer.

Variable	Predictive Result	Area under the Curve (AUC)	Confidence Interval (CI)
SMURF1	Tumor tissue vs normal tissue	0.931	0.902-0.960
SMURF2	Tumor tissue vs normal tissue	0.940	0.911-0.969

The correlation between the expression levels of SMURF1 and SMURF2 was examined. **Figure 2B** shows a positive correlation between their expression levels (rPearson=0.491, *P*<0.001, **Figure 2B**), suggesting that SMURF1 and SMURF2 may be subject to simultaneous transcriptional regulation.

### 3.4 Inhibition of SMURF1 inhibits the proliferation and migration of pancreatic cancer cells

**Figure 3:**
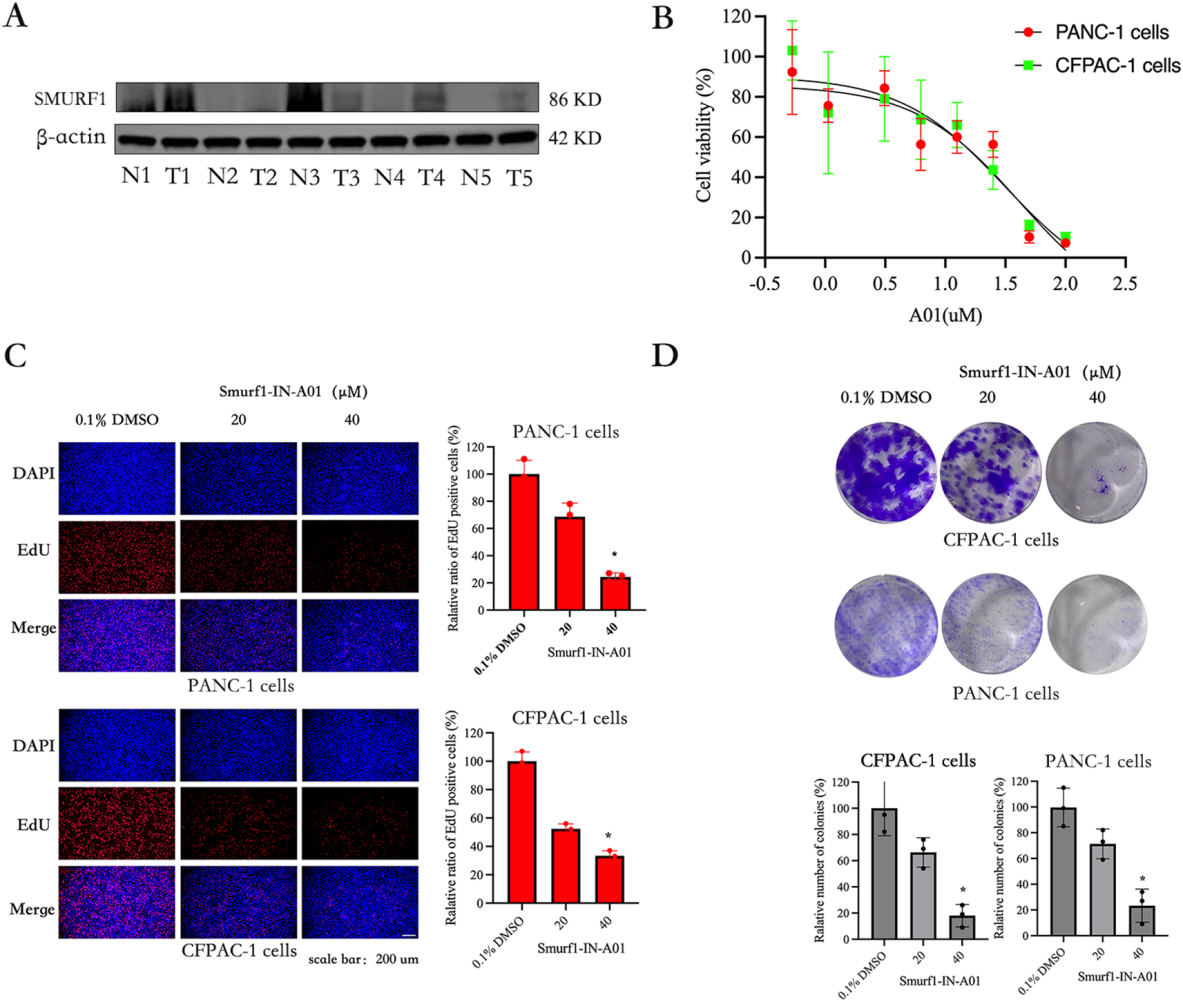
Smurf1-IN-A01 suppressed the proliferation of pancreatic cancer cell. **(A)** Expression of smurf1 in pancreatic cancer and adjacent tissues. **(B)** Pancreatic cancer cells were treated with different concentrations of Smurf1-IN-A01 (0-100) for 72 hours, and the survival rate was detected by CCK8 test. **(C)** Measurement of anti-proliferation effects of Smurf1-IN-A01 using EdU incorporation assay, scale bar: 200 μm. **(D)** Cell proliferation ability was assessed through colony formation assay following downregulation of Smurf1, scale bar: 200 μm. *P < 0.05, versus the control.

Extensive database analyses suggest that high SMURF1 expression may be a poor prognostic indicator for pancreatic cancer and a possible target for the treatment of pancreatic cancer. Five pancreatic cancer tissue samples confirmed by pathology were used to detect the difference in the expression of SMURF1 between pancreatic cancer tissue and paracancerous tissue. The results showed that the expression of SMURF1 in some tissues of pancreatic cancer patients was higher than that in adjacent tissues. Due to the small sample size, the conclusion was not universal (as shown in **Figure 3A**). To investigate the function of SMURF1 in the proliferation and migration of pancreatic cancer cells, SMURF1 was inhibited using the Smurf1-IN-A01 inhibitor, and the inhibitory effects on pancreatic cancer cells were verified using the *in vitro* CCK-8 assay, EdU assay, colony formation assay and scratch assay.

The CCK-8 kit assay was used to evaluate the effect of Smurf1-IN-A01 in pancreatic cancer cells. The results showed that Smurf1-IN-A01 could inhibit the growth of PANC-1 (IC50: 41μM) and CFPAC-1 (IC50: 31μM) cells in a dose-dependent manner (**Figure 3B**). Indicating that Smurf1-IN-A01 might be a potential SMURF1 targeted drug for pancreatic cancer. Based on this result, we selected PANC-1 and CFPAC-1 cells in the following experiments, with Smurf1-IN-A01 concentrations set at 20 μM and 40 μM concentration gradients.

PANC-1 and CFPAC-1 cells were treated with Smurf1-IN-A01 to confirm the inhibitory effect of SMURF1 inhibition using the EdU assay. As illustrated in **Figure 3C**, the percentage of EdU-positive cells in the Smurf1-IN-A01-treated groups were significantly lower compared to the control groups of PANC-1 and CFPAC-1 cells. The percentage of EdU-positive cells in PANC-1 and CFPAC-1 cells decreased to 24% and 34%, respectively, after treatment with 40 μM Smurf1-IN-A01. These results demonstrate that SMURF1 inhibition notably hindered the growth of pancreatic cancer cells.

The effect of Smurf1-IN-A01 on the colony forming ability of PANC-1 and CFPAC-1 cells was evaluated using a colony formation assay to observe the long-term inhibitory effect of SMURF1 inhibition on the proliferation of pancreatic cancer cells. As shown in **Figure 3D**, SMURF1 inhibition significantly inhibited the colony formation in PANC-1 and CFPAC-1 cells, with the number of colonies formed in the two types of cells reduced by 77% and 82%, respectively, after treatment with 40 μM Smurf1-IN-A01 compared to the control group. The data demonstrate that SMURF1 inhibition significantly impeded the colony forming ability of pancreatic cancer cells.

**Figure 4:**
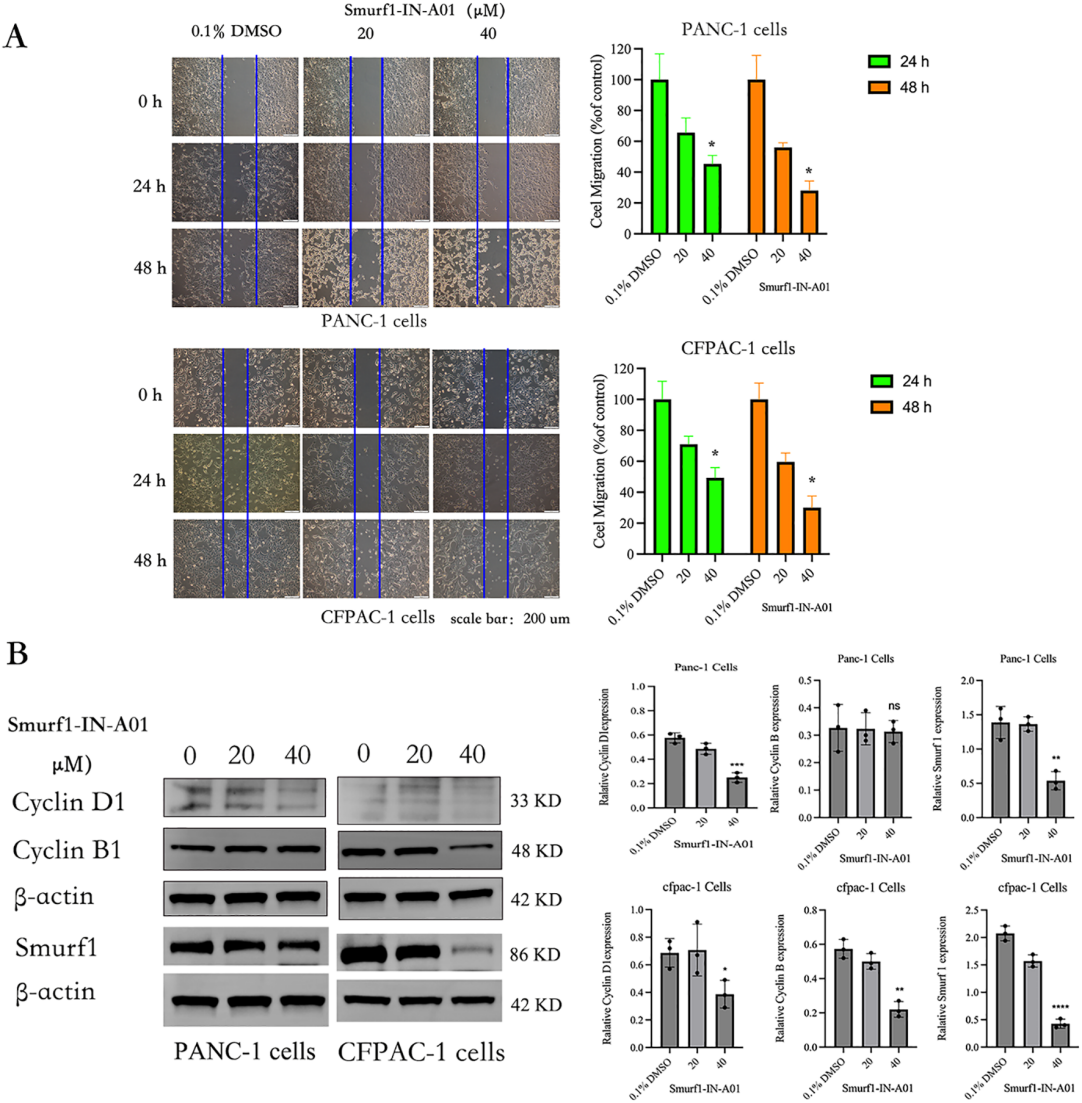
Smurf1-IN-A01 suppressed the migration of pancreatic cancer cell. **(A)** The impact of Smurf1-IN-A01 on the migration of PANC-1 and CFPAC-1 cells was evaluated via a wound-healing assay. The quantity of proliferating or migrating cells was standardized to the control. The outcomes are presented as the mean ± SEM of three replications, *P < 0.05, versus the control. **(B)** The effect of Smurf1-IN-A01 on cell cycle proteins. **P < 0.01, versus the control; ***P < 0.001, versus the control; ****P < 0.0001, versus the control. “ns”, no significance.

As shown in the **Figure 4A**, the results of the *in vitro* scratch assay showed that at 24 and 48 hours after scratching, the control group healed significantly faster than the Smurf1-IN-A01 group. Statistical analysis revealed a decrease in the migration rate of PANC-1 cells by around 55% and 72% at 24 and 48 hours, respectively, after inhibition of SMURF1. The migration rate of CFPAC-1 cells decreased by around 51% and 70% at 24 and 48 hours after cell scratching, respectively. The findings suggest that inhibition of SMURF1 considerably reduced the migration capability of pancreatic cancer cells.

The effects of Smurf1-IN-A01 on the levels of SMURF1 and cell cycle proteins Cyclin D1 and Cyclin B were analyzed by Western blotting (**Figure 4B**). Compared with the control group, the expression of SMURF1 in pancreatic cancer cells treated with Smurf1-in-A01 decreased (*P*<0.05). After treatment with 40μm Smurf1-in-A01, the expression of Cyclin D1 decreased in both PANC-1 (*P*<0.05) and CFPAC-1 (*P*<0.05) cells, while the expression of Cyclin B decreased in CFPAC-1 (*P*<0.05) cells but did not show any significant changes in PANC-1 (ns) cells. We speculate that inhibition of SMURF1 may inhibit the growth of pancreatic cancer cells by affecting cell cycle.

## 4 Discussion

In this study, SMURF1 and SMURF2 were found to be upregulated in pancreatic cancer, which is similar to the findings of Longtao Yang et al. (22). Additionally, their expression was found to correlate with the clinical stage of pancreatic cancer (**Table 1**), so it is postulated that increased expression of SMURF1 and SMURF2 may have prognostic value in identifying patients with a poor outcome (**Tables 1**, **2**; **Figure 1**). Co-expression analysis confirmed that SMURF1 and SMURF2 were highly correlated and co-regulated in pancreatic cancer. In terms of the diagnostic value for predicting tumors, both SMURF1 and SMURF2 showed high predictive accuracy (AUC = 0.931 and AUC = 0.940, respectively). Analysis of clinical and molecular characteristics revealed that differential expression of SMURF1 was associated with various parameters such as T stage, pathological stage, histological grade, and clinical outcomes. On the other hand, differential expression of SMURF2 was associated with histological grade, and the higher the clinical stage or disease progression, the higher the SMURF expression level (**Table 1**). These results indicate that a high level of SMURF expression is significantly associated with a higher stage of pancreatic cancer. But the amount of data we collected is relatively small, we will need to gather more data in the future to refine our viewpoint.

Recent studies have shown a negative correlation between Smurf1 expression levels and survival rates in gastric cancer and renal clear cells (19, 25). In the survival analysis, it was found that high expression of SMURF1 adversely affected OS and PFI, while high expression of SMURF2 only adversely affected PFI (**Table 2**). The proportion of high expression of smurf is higher than that of low expression in deceased patients or patients with disease progression. The results from the Cox regression analysis demonstrated that an elevated expression of SMURF2 had a negative correlation with the survival time of patients with pancreatic cancer (**Figure 1**), which is of great importance for predicting patient survival. Comprehensive analysis revealed that high expression of SMURF could be used as a poor prognostic marker for pancreatic tumors.

Recently, it has been reported that knocking out SMURF1 can reduce tumorigenesis in gastric cancer, prostate cancer, and ovarian cancer (19, 26, 27). SMURF1 may represent a potential target for the treatment of pancreatic cancer. To verify the role of SMURF1 in pancreatic cancer, SMURF1 specific inhibitor Smurf1-IN-A01 was selected to verify the effect of inhibiting SMURF1 on the migration and proliferation of PANC-1 and CFPAC-1 cells. Our results indicate that Smurf1-IN-A01 is an effective inhibitor of proliferation and migration of pancreatic cancer cells. It is inferred that this may be achieved by affecting the cell cycle to inhibit tumor cell growth. In conclusion, our results show that inhibition of SMURF1 can inhibit the proliferation and migration of pancreatic cancer cells, and Smurf1-IN-A01 is a promising candidate drug for the treatment of pancreatic cancer.

It has been shown that SMURF1 is a potential tumor promoting factor. Genomic hybridization analyses indicates that SMURF1 is a potential oncogenic factor in gastric cancer. Expression of SMURF1 was negatively associated with the survival of patients with gastric cancer and clear cell renal cell carcinoma (ccRCC). In addition, SMURF1 is required for the maintenance of stemness of tumor stem cells in head and neck squamous cell carcinoma (HNSCC) (28). Lin Fu et al. reported that SMURF1 can promote cancer metastasis by regulating various proteins that control cancer metastasis (23). Ubiquitination is essential for apoptosis signaling pathway, and SMURF1 may act as an apoptosis inhibitor through regulation of the ubiquitination process. These findings demonstrate that SMURF1 may be a potential therapeutic target for pancreatic cancer, providing new perspectives for evaluating the role of SMURF1 inhibitors in the clinical management of human pancreatic cancer.

## 5 Conclusion

In conclusion, our results indicate that the high expression of SMURF may be a biomarker for poor prognosis of pancreatic cancer. *In vitro* experiments show that SMURF1 plays an important role in the proliferation and migration of pancreatic cancer cells. We speculate that SMURF1 may promote apoptosis of pancreatic cancer cells and affect cell cycle progression by inhibiting the ubiquitin process of cells, thus inhibiting cell proliferation and migration. Therefore, SMURF has significance in predicting the clinical stage and prognosis of pancreatic cancer, and smurf1 can be a potential target for the treatment of pancreatic cancer. However, the number of samples included in our study is relatively small at present, and more experiments need to be conducted, including *in vivo* experiments to validate our results. The mechanism by which SMURF1 leads to poor cancer prognosis requires further research.

## Data Availability

The datasets presented in this study can be found in online repositories. The names of the repository/repositories and accession number(s) can be found below: https://portal.gdc.cancer.gov/.
